# Fat Intake Modifies the Association between Restricted Carbohydrate Diets and Prevalent Cardiometabolic Diseases among Adults in the United States: National Health and Nutrition Examination Survey, 1999–2018

**DOI:** 10.1016/j.cdnut.2022.100019

**Published:** 2022-12-23

**Authors:** Corina Kowalski, Dakota Dustin, LuAnn K. Johnson, Martha A. Belury, Zach Conrad

**Affiliations:** 1 College of Arts & Sciences, William & Mary, Williamsburg, VA, USA; 2 Department of Human Sciences, Ohio State University, Columbus, OH, USA; 3 Independent Contractor, Warren, MN, USA; 4 Department of Kinesiology, William & Mary, Williamsburg, VA, USA

**Keywords:** restricted carbohydrate, cardiometabolic disease, heart disease, fat, NHANES, popular diet

## Abstract

**Background:**

Cardiometabolic diseases (CMDs), which include heart disease, stroke, and diabetes, account for over one-third of the mortality burden in the United States annually. Nearly one-half of all deaths from CMD are attributable to suboptimal diet quality, and many Americans are turning to special diets for general health improvement. Among the most popular of these diets restrict daily carbohydrate intake to <45% of energy, yet their association with CMD is not well understood.

**Objectives:**

This study evaluated the association between restricted carbohydrate diets and prevalent CMD, stratified by fat intake.

**Methods:**

Dietary and CMD data were retrieved from 19,078 participants aged ≥20 y in the National Health and Nutrition Examination Survey, 1999–2018. The National Cancer Institute methodology was used to assess usual dietary intake.

**Results:**

Compared to participants that met recommendations for all macronutrients, those that consumed restricted carbohydrate diets were 1.15 (95% CI: 1.14, 1.16) times as likely to have CMD; and those that met recommendations for carbohydrates, but not all macronutrients, were 1.02 (95% CI: 1.02, 1.03) times as likely to have CMD. Higher intakes of saturated and polyunsaturated fat were associated with greater prevalence of CMD in restricted and recommended carbohydrate intake groups. Higher intake of monounsaturated fat was associated with lower prevalence of CMD among participants that met carbohydrate, but not all macronutrient, recommendations.

**Conclusions:**

To our knowledge, this is the first nationally representative study to evaluate the relationship between carbohydrate restriction and CMD, stratifying by fat intake. Greater efforts are needed to understand longitudinal relationships between carbohydrate restriction and CMD.

## Introduction

Heart disease remains the leading cause of death in the United States [1], currently accounting for >650,000 deaths per year [2]. Combined with stroke and diabetes, which respectively represent the fifth and seventh leading causes of death, cardiometabolic diseases (CMDs) accounted for >30% of the mortality burden in adults in the United States in 2019 [1]. Despite a reduction in the death rate attributable to heart disease and stroke since 1980 [2] the absolute mortality burden and an increase in the prevalence of diabetes over the past 30 y warrants continued attention [2, 3].

Suboptimal diet quality represents the leading risk factor for death from all causes [4] and accounts for approximately one-half of all deaths from CMD [5]. Low-quality diet patterns are typically characterized by high amounts of calories, added sugar, and sodium, and low amounts of fruits, vegetables, whole grains, and nuts and seeds [5]. Low-quality diets are also closely linked with obesity, which has increased in prevalence from <30% in 1999–2000 to >40% in 2017–2018 [6] and represents the fourth leading risk factor for death in the United States [4]. Despite moderate improvements in adherence to food-based dietary guidance since 1999, <5% of the US population meets these recommendations [7, 8], leaving consumers with a substantial but preventable risk to their health.

Despite the failure to meet key dietary recommendations, Americans demonstrate an avid interest in dieting. Approximately 43% of consumers reported following a diet in 2020 for weight loss and/or general health improvement, an increase of 5 percentage points over the previous year [9]. Key among types of weight loss diets being used are carbohydrate restricted diets, which typically fall below the acceptable macronutrient distribution range of 45%–65% energy (%en) from carbohydrates [10]. Despite the popularity of carbohydrate restricted diets, the evidence to support their benefits for long-term cardiometabolic health is inconsistent and may even be harmful [11]. Recent prospective cohort studies have demonstrated that restricted carbohydrate diets were associated with similar or increased risk of coronary artery calcium progression compared with higher carbohydrate diets [12, 13], and a recent meta-analysis of prospective cohort studies demonstrated elevated risk of mortality from all causes and cardiovascular disease (CVD) [14]. Nationally representative prospective studies using data collected by the National Health and Nutrition Examination Survey (NHANES) linked to follow-up data from public mortality files showed mixed results, with one study showing greater mortality risk associated with lower carbohydrate diets [14] and another showing no association [15]. Furthermore, other studies have reported no association between lower carbohydrate diets and incident CVD, myocardial infarction, stroke, or CVD mortality [[15], [16], [17]].

Variability of fat quantity and fat class in restricted carbohydrate diets may affect their association with CMD [11, 16]. For example, evidence supports a positive relationship between intake of saturated fatty acids (SFAs) and CVD risk [17], although the strength of this relationship remains under debate [18]. Strong and consistent evidence also supports an association between greater intake of polyunsaturated fatty acids (PUFAs; particularly linoleic acid: 18:2n6, eicosapentaenoic acid: 20:5n3, and docosahexaenoic acid: 22:6n3) and reduced risk of heart disease [[19], [20], [21]], type 2 diabetes, and CMD risk factors [[22], [23], [24]]. The association between monounsaturated fatty acid (MUFA) intake and CMD appears to be marginal [25, 26], but replacing SFA with PUFA or MUFA is associated with improved health outcomes [27]. A lack of accounting for differences in fatty acid composition of lower carbohydrate diets may contribute to inconsistent associations with CMD. Further research is needed to inform clinical practice guidelines and public health recommendations to help consumers make informed decisions about their food choices to improve CMD outcomes.

To the best of our knowledge, no studies have evaluated the relationship between restricted carbohydrate diets and prevalent CMD, stratified by fat intake, among a nationally representative sample of US adults. To fill this research gap, this study used 20 y (1999–2018) of data on usual dietary intake from NHANES to evaluate the association between *1*) restricted carbohydrate diets and prevalent CMD, additionally stratified by intake of 2) SFA, 3) MUFA, 4) PUFA, and 5) total fat.

## Methods

### Data acquisition

Individual-level data on food intake, nutrient intake from foods and supplements, sociodemographics, and prevalent heart disease, stroke, and diabetes were acquired from the NHANES, 1999–2018. NHANES uses a stratified, clustered, multistage sampling design, and trained staff collect data from approximately 5000 noninstitutionalized participants per year using in-person surveys, physical examinations, and laboratory tests. Some population groups are oversampled to increase reliability and precision for subgroup analysis [28]. Data are released in 2-y cycles [29]. Trained interviewers collect dietary data from participants using an in-person 24-h dietary recall, and a subsequent recall is administered by telephone 3 to 10 d later on ∼80% of the sample [30]. The computer-assisted automated multiple-pass method is used to minimize respondent burden and increase reliability and validity [31, 32]. The present study is a secondary analysis of publicly available and deidentified data and was deemed exempt from human studies ethical review by the institutional review board at William & Mary. Preregistration for this study can be found elsewhere [33].

### Diet categorization

The National Cancer Institute’s (NCI) usual intake methodology was used to assess usual intake of foods and nutrients [34]. The NCI method uses data from two 24-h recalls collected from most participants to estimate within-person variation of the entire sample using the SAS macros MIXTRAN, DISTRIB, and INDIVINT [[35], [36], [37]]. MIXTRAN fits a nonlinear mixed-effects model to repeated 24-h recalls, and parameter estimates are passed to DISTRIB to estimate the distribution of usual intake in the population of interest; these distributions were used to categorize tertiles of fat intake. Parameter estimates from MIXTRAN are also passed to INDIVINT, which estimates intake at the individual level.

Participants were primarily categorized (diet categorization 1) as restricted carbohydrate (<45%en), recommended carbohydrate only (45%–65%en), and those that met all macronutrient recommendations (total carbohydrate, 45%–65%en; total fat, 20%–35%en; total protein, 10–35%en; α-linolenic acid, 0.6%–1.2%en; and linoleic acid, 5%–10%en). Recommended carbohydrate only was defined as those meeting carbohydrate recommendations but not meeting recommendations for protein and/or fat. These categorizations are consistent with those used by the Nutrition and Lifestyle Task Force of the National Lipid Association [11] and the Food and Nutrient Board of the Institute of Medicine, National Academy of Sciences (Dietary Reference Intakes) [38]. To investigate the effects of fat class, 4 additional categorization schemes were constructed by stratifying each of these primary categories by intake tertiles of SFA (diet categorization 2), MUFA (diet categorization 3), PUFA (diet categorization 4), and total fat (diet categorization 5).

### Outcome ascertainment

Diagnostic criteria for CMD were based on standard measures from the American Heart Association [2]. Prevalent diabetes was identified by self-report of physician diagnosis (DIQ010), fasting plasma glucose level of ≥126 mg/dL (LBXGLU), or self-report of current prescription drug treatment (DIQ050 or DIQ070). Participants with a history of stroke (MCQ160F) were identified by self-report of physician diagnosis. Prevalent coronary heart disease (CHD) was identified by self-report of physician diagnosis of myocardial infarction (MCQ160C or MCQ160E) or angina (MCQ160D) or taking angina medications nitroglycerin, isosorbide dinitrate, or isosorbide mononitrate (RDXDRUG), and undiagnosed angina was based on the Rose questionnaire (CDQ001–CDQ009G). Participants with CHD or a history of stroke were identified as having CVD; therefore, CMD included those with CVD or diabetes.

### Statistical analyses

Multivariable logistic regression models assessed the association between diet patterns and CMD, CVD, CHD, stroke, and diabetes. All models were adjusted for age (20–30 y, 31–50 y, 61–70 y, or ≥71 y), sex (male or female), education (>high school, high school or equivalent, some college, or college graduate), race-ethnicity (non-Hispanic White, non-Hispanic Black, Mexican-American, or other), income-to-poverty ratio (<0.75, 0.75–1.30, 1.31–1.99, 2.00–3.99, or ≥4.00), physical activity (sedentary, moderate, or vigorous), smoking status (<100 cigarettes in lifetime, ≥100 cigarettes in lifetime but not current smoker, ≥100 cigarettes in lifetime and currently smoke some days, or ≥100 cigarettes in lifetime and currently smoke everyday), BMI (kg/m^2^; <18.5, 18.5 to <25, 25 to <30, and ≥30), energy (continuous), refined grains (continuous), added sugars (continuous), fiber (continuous), total protein (%en, continuous), and survey cycle (continuous). Missing values for each covariate were included as a dummy indicator to preserve sample size.

Models stratified by saturated fat intake (diet categorization 2) divided participants based on tertiles of high (>12.5%en), moderate (11.4%–12.5%en), and low (<11.4%en) intake of saturated fat. These models were additionally adjusted for intake of monounsaturated fat (continuous), polyunsaturated fat (continuous) and palmitic acid (continuous). Models stratified by monounsaturated fat intake (diet categorization 3) divided participants based on tertiles of high (>14%en), moderate (12.9%–14%en), and low (<12.9%en) intake of monounsaturated fat. These models were additionally adjusted for intake of oleic acid (continuous), saturated fat (continuous), and polyunsaturated fat (continuous). Models stratified by polyunsaturated fat intake (diet categorization 4) divided participants based on tertiles of high (>8.5%en), moderate (7.6%–8.5%en), and low (<7.6%en) intake of polyunsaturated fat. These models were additionally adjusted for intake of saturated fat(continuous), monounsaturated fat (continuous), linoleic acid (continuous), α-linolenic acid (continuous), docosahexaenoic acid (continuous), and eicosapentaenoic acid (continuous). Models stratified by total fat intake (diet categorization 5) divided participants based on tertiles of high (>38.7%en), moderate (36.1%–38.7%en), and low (<36.1%en) intake of total fat. These models were additionally adjusted for unsaturated-to-saturated fat ratio (continuous). For each diet categorization, models were adjusted for these additional covariates iteratively to investigate the confounding effect of each covariate separately.

Statistical significance was set at *P* value of < 0.05 with Bonferroni adjustment for multiple comparisons. Standard errors were estimated using the balanced repeated replication method while accounting for the multistage probability sampling design of NHANES. CIs and *P* values for odds ratios were calculated using the NCI macro BRR_PVALUE_CI (v1.1) [34]. Stata 16.1 (StataCorp) was used for data management and SAS 9.4 (SAS Institute) was used for all analyses.

## Results

Data were retrieved from 96,766 individuals from NHANES 1999–2018 (Table 1). Exclusion criteria removed participants <20 y (*n* = 44,368), with incomplete or unreliable dietary data or <8.5 h fasting prior to blood draw (*n* = 26,546), with incomplete data on cardiometabolic health status or pregnant or breastfeeding (*n* = 6,769), or consumed >65%en from carbohydrates (*n* = 5). The final sample included 19,078 participants, including 4424 that consumed restricted carbohydrate diets defined as <45%en from carbohydrates. The group of participants who reported 45%–65%en from carbohydrates was divided into 2 subgroups: 6628 met carbohydrate but not all macronutrient recommendations, and 8026 met recommendations for all macronutrients.

**TABLE 1:** Participant characteristics

Characteristic	Restricted carbohydrate (*n* = 4424)	Recommended carbohydrate (*n* = 6628)	Recommended all macronutrients (*n* = 8026)	*P*^1^
Age, y	47.8 (47.1–48.5)	48.2 (47.5–48.9)	47.6 (47–48.3)	0.307
Income-to-poverty ratio	3.3 (3.2–3.4)	3.0 (2.9–3.0)	2.9 (2.8–3.0)	<0.001
Female, %	42.9 (40.9–45.0)	52.0 (50.3–53.7)	53.7 (52.1–55.3)	<0.001
Education, %				<0.001
Less than high school	13.9 (12.5–15.4)	17.4 (16.1–18.7)	18.3 (17–19.7)	
High school or equivalent	23.0 (21.2–24.8)	26.5 (24.9–28.1)	23.3 (21.5–25.1)	
Some college	31.3 (29.3–33.4)	32.7 (31.0–34.5)	29.7 (28.3–31.1)	
College graduate	31.8 (29.4–34.3)	23.3 (21.4–25.4)	28.7 (26.6–31)	
Race-ethnicity, %				<0.001
Non-Hispanic White	74.9 (72.6–77.0)	71.8 (69.3–74.2)	65.4 (63.0–67.7)	
Non-Hispanic Black	9.7 (8.5–11.0)	10.9 (9.5–12.4)	10.0 (8.9–11.2)	
Mexican-American	5.8 (4.8–7.0)	8.3 (7.0–9.6)	8.9 (7.8–10.3)	
Other	9.7 (8.4–11.1)	9.1 (8.0–10.3)	15.7 (14.2–17.3)	
Macronutrient intake %en				
Carbohydrate	41.5 (41.4–41.7)	49.1 (49.0–49.2)	51.4 (51.3–51.5)	<0.001
Fat	37.1 (36.9–37.2)	35.2 (35.1–35.3)	32.6 (32.5–32.7)	<0.001
Protein	16.3 (16.3–16.4)	15.1 (15.0–15.1)	15.4 (15.4–15.5)	<0.001
Alcohol, g	15.6 (15.1–16.1)	6.9 (6.6–7.2)	7.9 (7.6–8.2)	<0.001
Smoking status %				<0.001
<100 cigarettes/lifetime	44.9 (42.6–47.3)	52.9 (50.8–54.9)	56.5 (54.7–58.3)	
≥100 cigarettes/lifetime, currently nonsmoking	30.3 (28.3–32.5)	25.4 (23.7–27.2)	24.6 (23.2–26.1)	
≥100 cigarettes/lifetime, currently smoking some days	4.3 (3.5–5.2)	3.0 (2.5–3.5)	3.4 (2.8–4.1)	
≥100 cigarettes/lifetime, currently smoking every day	20.4 (18.5–22.4)	18.7 (17.1–20.3)	15.5 (14.1–16.9)	
Physical activity, %				<0.001
Sedentary	23.7 (21.9–25.6)	29.1 (27.5–30.9)	24.4 (22.9–25.9)	
Moderate	35.0 (33.0–37.0)	34.4 (32.6–36.2)	35.7 (34.0–37.5)	
Vigorous	41.3 (39.2–43.5)	36.5 (34.8–38.2)	39.8 (37.9–41.8)	

1 Differences between diet categories assessed using Wald tests with *P* < 0.05 for continuous variables (age, income-to-poverty ratio, macronutrient intake, and alcohol), and differences between diet categories assessed using χ^2^ tests with *P* < 0.05 for categorical variables (female, education, race-ethnicity, smoking status, and physical activity).

Mean carbohydrate intake as a percentage of total energy was 42%en, 49%en, and 51%en among participants that consumed restricted carbohydrate diets, those that met carbohydrate but not all macronutrient recommendations, and those that met all macronutrient recommendations, respectively (Table 1). Participants that consumed restricted carbohydrate diets also had greater intake of fat (37%en) and protein (16%en) than other groups. Participants that consumed a restricted carbohydrate diet had greater income-to-poverty ratios (3.3), were more likely to be college graduates (32%), non-Hispanic White (75%), and physically active (41%), but were less likely to be women (43%) and to have smoked <100 cigarettes in their lifetime (45%). Mean age was similar across the groups (48 y).

Compared with participants that met all macronutrient recommendations (Table 2), those that consumed restricted carbohydrate diets were 1.15 (95% CI: 1.14, 1.16) times as likely to have CMD (*P* < 0.001), and those that consumed only the recommended amount of carbohydrates were 1.02 (95% CI: 1.02, 1.03) times as likely to have CMD (*P* < 0.001). Odds ratios for specific end points (CVD, CHD, stroke, and diabetes) are presented in Supplemental Table 1.

**TABLE 2:** Association between restricted carbohydrate diets and cardiometabolic disease (*n* = 19,078)

Diet category	*n*	OR (95% CI)^1^	*P*^2^
Restricted carbohydrate^3^	4424	1.15 (1.14, 1.16)	<0.001
Recommended carbohydrate^4^	6628	1.02 (1.02, 1.03)	<0.001
Recommended all macronutrients^5^	8026	Reference	

CI, confidence interval; OR, odds ratio.

1 Adjusted for age, sex, education, race/ethnicity, income-to-poverty ratio, physical activity, smoking status, energy, refined grains, added sugars, fiber, protein, survey wave, alcohol, and BMI

2 Wald test with Bonferroni correction for multiple comparisons

3 Restricted carbohydrate (<45%en carbohydrate)

4 Recommended carbohydrate (45%–65%en carbohydrate)

5 Recommended all macronutrients (total carbohydrate, 45%–65%en; total fat, 20%–35%en; total protein, 10%–35%en; α-linolenic acid, 0.6%–1.2%en; and linoleic acid, 5%–10%en)

Compared with those that met all macronutrient recommendations and were in the lowest tertile of saturated fat consumption (<11.4%en from SFA; Table 3), those that consumed restricted carbohydrate diets and high amounts of saturated fat (>12.5%en) were 1.26 (95% CI: 1.25, 1.28) times more likely to have CMD, those that consumed moderate amounts of saturated fat (11.4%–12.5%en) were 1.22 (95% CI: 1.21, 1.22) times more likely, and those within the lowest intake tertile of saturated fat were 1.04 (95% CI: 1.03, 1.05) times as likely (*P* < 0.001 for all comparisons). Higher intake of saturated fat was associated with higher likelihood of prevalent CMD among those that met only recommended carbohydrate intake and those that met all macronutrient recommendations but were of lower magnitude. Further adjustment for %en from PUFA and MUFA (fully adjusted model) as well as stearic acid (supplemental model, Supplemental Table 2) modestly attenuated these findings, but did not completely alter key findings (*P* < 0.001 for all comparisons).

**TABLE 3:** Association between restricted carbohydrate diet stratified by saturated fat intake and cardiometabolic disease (*n* = 19,078)

Diet category	n	Base model^1^		Fully adjusted model^2^	
		OR (95% CI)	P^3^	OR (95% CI)	P^3^
Restricted carbohydrate^4^	4424				
High saturated fat^5^	1386	1.26 (1.25, 1.28)	<0.001	1.20 (1.19, 1.21)	<0.001
Moderate saturated fat^6^	1481	1.22 (1.21, 1.22)	<0.001	1.15 (1.14, 1.16)	<0.001
Low saturated fat^7^	1557	1.04 (1.03, 1.05)	<0.001	1.00 (0.98, 1.01)	0.897
Recommended carbohydrate^8^	6628				
High saturated fat^5^	1165	1.04 (1.03, 1.05)	<0.001	1.02 (1.01, 1.03)	<0.001
Moderate saturated fat^6^	2392	1.06 (1.06, 1.07)	<0.001	1.03 (1.03, 1.04)	<0.001
Low saturated fat^7^	3071	1.04 (1.04, 1.05)	<0.001	1.02 (1.02, 1.03)	<0.001
Recommended all macronutrients^9^	8026				
High-moderate saturated fat^5–6^	1412	1.11 (1.11, 1.12)	<0.001	1.11 (1.11, 1.12)	<0.001
Low saturated fat^7^	6614	Reference		Reference	

CI, confidence interval; OR, odds ratio.

1 Adjusted for age, sex, education, race/ethnicity, income-to-poverty ratio, physical activity, smoking status, energy, refined grains, added sugars, fiber, protein, survey wave, BMI, and alcohol

2 Adjusted for age, sex, education, race/ethnicity, income-to-poverty ratio, physical activity, smoking status, energy, refined grains, added sugars, fiber, protein, survey wave, alcohol, total monounsaturated fatty acids, polyunsaturated fatty acids, and BMI

3 Wald test with Bonferroni correction for multiple comparisons

4 Restricted carbohydrate (<45%en carbohydrate)

5 High saturated fat (>12.5%en)

6 Moderate saturated fat (11.4%–12.5%en)

7 Low saturated fat (<11.4%en)

8 Recommended carbohydrate (45%–65%en carbohydrate)

9 Recommended all macronutrients (total carbohydrate, 45%–65%en; total fat, 20%–35%en; total protein, 10%–35%en; α-linolenic acid, 0.6%–1.2%en; and linoleic acid, 5%–10%en)

Diets were additionally stratified for MUFA intake, separating participants into tertiles of high (>14.0%en), moderate (12.9%–14.0%en), and low (<12.9%en) MUFA intake. Compared with a reference group that met all macronutrient recommendations and fell within the lowest tertile of MUFA intake (Table 4), prevalent CMD was 1.26 (95% CI: 1.25, 1.27), 1.16 (95% CI: 1.16, 1.17), and 0.91 (95% CI: 0.89, 0.92) times as likely among carbohydrate restricted participants that consumed within the high, moderate, and low tertiles, respectively. Variable associations were observed between levels of MUFA intake and CMD among participants who met only carbohydrate recommendations. Among participants who met all macronutrient recommendations, higher intake of MUFA was associated with reduced likelihood of having CMD. After further adjustment for %en from SFA and PUFA (fully adjusted model), greater intake of MUFA was associated with lower likelihood of having CMD among participants who met only carbohydrate recommendations and those who met all macronutrient recommendations, whereas the opposite relationship was demonstrated among participants that consumed restricted carbohydrate diets. Additional adjustment for oleic acid intake (Supplemental Table 3) attenuated all associations but did not alter key findings (*P* < 0.001 for all comparisons).

**TABLE 4:** Association between restricted carbohydrate diet stratified by monounsaturated fat intake and cardiometabolic disease (*n* = 19,078)

Diet category	*n*	Base model^1^		Fully adjusted model^2^	
		OR (95% CI)	*P*^3^	OR (95% CI)	*P*^3^
Restricted carbohydrate^4^	4424				
High monounsaturated fat^5^	1512	1.26 (1.25, 1.27)	<0.001	1.16 (1.15, 1.17)	<0.001
Moderate monounsaturated fat^6^	1535	1.16 (1.16, 1.17)	<0.001	1.08 (1.06, 1.09)	<0.001
Low monounsaturated fat^7^	1377	0.91 (0.89, 0.92)	<0.001	0.87 (0.85, 0.88)	<0.001
Recommended carbohydrate^8^	6628				
High monounsaturated fat^5^	612	0.92 (0.91, 0.93)	<0.001	0.88 (0.87, 0.88)	<0.001
Moderate monounsaturated fat^6^	2783	1.03 (1.02, 1.03)	<0.001	0.98 (0.97, 0.98)	<0.001
Low monounsaturated fat^7^	3233	1.03 (1.03, 1.04)	<0.001	1.01 (1.01, 1.01)	<0.001
Recommended all macronutrients^9^	8026				
Moderate-high monounsaturated fat^5-6^	478	0.82 (0.82, 0.82)	<0.001	0.813 (0.81, 0.82)	<0.001
Low monounsaturated fat^7^	7548	Reference		Reference	

1 Adjusted for age, sex, education, race/ethnicity, income-to-poverty ratio, physical activity, smoking status, energy, refined grains, added sugars, fiber, protein, survey wave, alcohol, and BMI

2 Adjusted for age, sex, education, race/ethnicity, income-to-poverty ratio, physical activity, smoking status, energy, refined grains, added sugars, fiber, protein, survey wave, alcohol, saturated fatty acids, monounsaturated fatty acids, and BMI

3 Wald test with Bonferroni correction for multiple comparisons

4 Restricted carbohydrate (<45%en carbohydrate)

5 High monounsaturated fat (>14%en)

6 Moderate monounsaturated fat (12.9%–14%en)

7 Low monounsaturated fat (<12.9%en)

8 Recommended carbohydrate (45%–65%en carbohydrate)

9 Recommended all macronutrients (total carbohydrate, 45%–65%en; total fat, 20%–35%en; total protein, 10%–35%en; α-linolenic acid, 0.6%–1.2%en; and linoleic acid, 5%–10%en)

Compared with those that met all macronutrient recommendations and had a relatively low PUFA intake (Table 5), participants in the restricted carbohydrate diet group that consumed within the high (>8.5%en), moderate (7.6%–8.5%en), and low (<7.6%en) tertiles of PUFA were 1.18 (95% CI: 1.17, 1.19), 1.14 (95% CI: 1.14, 1.15), and 1.06 (95% CI: 1.05, 1.07) times as likely to have CMD, respectively. Greater intake of PUFA was associated with an elevated likelihood of having CMD among participants who met only recommended carbohydrate intake but was associated with lower likelihood of having CMD among participants who met all macronutrient recommendations. Additional adjustment for %en from SFA and MUFA (fully adjusted model), as well as linolenic acid, α-linoleic acid, eicosapentaenoic acid, and docosapentaenoic acid (supplemental model, Supplemental Table 4) attenuated all associations but did not alter key findings (*P* < 0.001 for all comparisons).

**TABLE 5:** Association between restricted carbohydrate diet stratified by polyunsaturated fat intake and cardiometabolic disease (*n* = 19,078)

Diet category	*n*	Base model^1^		Fully adjusted model^2^	
		OR (95% CI)	*P*^3^	OR (95% CI)	*P*^3^
Restricted carbohydrate^4^	4424				
High polyunsaturated fat^5^	1477	1.18 (1.17, 1.19)	<0.001	1.11 (1.10, 1.12)	<0.001
Moderate polyunsaturated fat^6^	1502	1.14 (1.14, 1.15)	<0.001	1.06 (1.05, 1.07)	<0.001
Low polyunsaturated fat^7^	1445	1.06 (1.05, 1.07)	<0.001	0.98 (0.96, 0.99)	<0.001
Recommended carbohydrate^8^	6628				
High polyunsaturated fat^5^	1524	1.05 (1.05, 1.06)	<0.001	1.04 (1.03, 1.04)	<0.001
Moderate polyunsaturated fat^6^	2243	1.01 (1.01, 1.02)	<0.001	0.97 (0.96, 0.97)	<0.001
Low polyunsaturated fat^7^	2861	0.98 (0.98, 0.99)	<0.001	0.95 (0.95, 0.95)	<0.001
Recommended all macronutrients^9^	8026				
High-moderate polyunsaturated fat^5–6^	3259	0.97 (0.97, 0.97)	<0.001	0.99 (0.99, 0.99)	<0.001
Low polyunsaturated fat^7^	4767	Reference		Reference	

1 Adjusted for age, sex, education, race/ethnicity, income-to-poverty ratio, physical activity, smoking status, energy, refined grains, added sugars, fiber, protein, survey wave, alcohol, and BMI

2 Adjusted for age, sex, education, race/ethnicity, income-to-poverty ratio, physical activity, smoking status, energy, refined grains, added sugars, fiber, protein, survey wave, alcohol, saturated fatty acids, and monounsaturated fatty acids

3 Wald test with Bonferroni correction for multiple comparisons

4 Restricted carbohydrate (<45%en carbohydrate)

5 High polyunsaturated fat (>8.5%en)

6 Moderate polyunsaturated fat (7.6%–8.5%en)

7 Low polyunsaturated fat (<7.6%en)

8 Recommended carbohydrate (45%–65%en carbohydrate)

9 Recommended all macronutrients (total carbohydrate, 45%–65%en; total fat, 20%–35%en; total protein, 10%–35%en; α-linolenic acid, 0.6%–1.2%en; and linoleic acid, 5%–10%en)

Participants who consumed restricted carbohydrate diets and fell within high (>38.7%en), moderate (36.1%–38.7%en), and low (<36.1%en) tertiles of total fat intake (Table 6) were 1.40 (95% CI: 1.38, 1.41), 1.08 (95% CI: 1.08, 1.09), and 0.91 (95% CI: 0.90, 0.93) times more likely to have CMD compared with participants who met all macronutrient recommendations. Greater intake of total fat was associated with greater likelihood of prevalent CMD among participants who met only recommended carbohydrate intake. Additional adjustment for unsaturated-to-saturated fat ratio (fully adjusted model) did not attenuate these findings (*P* < 0.001 for all comparisons).

**TABLE 6:** Association between restricted carbohydrate diet stratified by total fat intake and cardiometabolic disease (*n* = 19,078)

Diet category	*n*	Base model^1^		Fully adjusted model^2^	
		OR (95% CI)	*P*^3^	OR (95% CI)	*P*^3^
Restricted carbohydrate^4^	4424				
High total fat^5^	1434	1.40 (1.38–1.41)	<0.001	1.41 (1.39–1.43)	<0.001
Moderate total fat^6^	1538	1.08 (1.08–1.09)	<0.001	1.09 (1.08–1.09)	<0.001
Low total fat^7^	1452	0.91 (0.90–0.93)	<0.001	0.92 (0.90–0.93)	<0.001
Recommended carbohydrate^8^	6628				
High total fat^5^	254	1.14 (1.13–1.16)	<0.001	1.15 (1.14–1.17)	<0.001
Moderate total fat^6^	2731	1.08 (1.07–1.09)	<0.001	1.08 (1.07–1.09)	<0.001
Low total fat^7^	3643	1.00 (1.00–1.00)	0.003	1.00 (1.00–1.00)	<0.001
Recommended all macronutrients^9^	8026	Reference		Reference	

1 Adjusted for age, sex, education, race/ethnicity, income-to-poverty ratio, physical activity, smoking status, energy, refined grains, added sugars, fiber, protein, survey wave, BMI, and alcohol

2 Adjusted for age, sex, education, race/ethnicity, income-to-poverty ratio, physical activity, smoking status, energy, refined grains, added sugars, fiber, protein, survey wave, BMI, unsaturated-to-saturated fat ratio, and alcohol

3 Wald test with Bonferroni correction for multiple comparisons

4 Restricted carbohydrate (<45%en carbohydrate–

5 High total fat (>38.7%en)

6 Moderate total fat (36.1%–38.7%en)

7 Low total fat (<36.1%en)

8 Recommended carbohydrate (45%–65%en carbohydrate)

9 Recommended all macronutrients (total carbohydrate, 45%–65%en; total fat, 20%–35%en; total protein, 10%–35%en; α-linolenic acid, 0.6%–1.2%en; and linoleic acid, 5%–10%en)

## Discussion

In this nationally representative study of nearly 20,000 individuals over a 20-y time period, those that consumed restricted carbohydrate diets (<45%en from carbohydrates) had a 15% higher likelihood of prevalent CMDs compared with those that met daily macronutrient recommendations. Higher intake of total fat or any class of fat was associated with elevated likelihood of having CMD among those that consumed restricted carbohydrate diets, and these findings were maintained after additional adjustment for dietary factors including refined grains, added sugar, fiber, protein, and alcohol. Similar findings were observed among those that consumed recommended carbohydrate diets for total fat and PUFA and SFA fat classes but were of lower magnitude. Among participants who met all macronutrient recommendations, higher intake of saturated fat was associated with greater likelihood of prevalent CMD, whereas higher intake of MUFA or PUFA was associated with lower likelihood of CMD.

To the best of our knowledge, no other studies have evaluated the association between prevalent CMD and restricted carbohydrate diets stratified by fat intakes among a nationally representative sample of US adults. Using NHANES data from 1999–2010 with follow-up until 2011, other studies have shown an elevated risk of mortality from CHD and stroke associated with lower carbohydrate diets of 40%–50%en from carbohydrates [14], which is consistent with the present study for CHD but not for stroke. However, a more recent analysis of NHANES data from 1999–2014 with follow-up until 2015 showed no association between lower carbohydrate diets and heart disease [15].

The present study demonstrated that greater intake of total or all fat classes was associated with elevated odds of having CMD among participants who consumed restricted carbohydrate diets compared with participants who met all macronutrient recommendations. Unexpectedly, there was a positive association between PUFA intake and CMD regardless of carbohydrate intake. A meta-analysis of randomized controlled trials found a 10% reduction in death caused by CHD for every 1 g/d of dietary SFA intake replaced by PUFAs, specifically α-linoleic acid [39]. The same meta-analysis, citing another controlled trial, reported a 12% decrease in sudden cardiac death for every 0.1% increase in energy from α-linoleic acid [39]. Much of the prior research substituted PUFA in place of SFA, not only increasing PUFA but simultaneously decreasing SFA [39]. It is possible that overall dietary fat intake, including enhanced intake of unsaturated fats, has a stronger association with CVD than the reduction of saturated fat intake alone[17]. The unexpected positive association between PUFA or MUFA intake and CMD prevalence in the current study may have been similarly affected by the presence of accompanying total fat, as 96% of those within the high PUFA tertile also consumed high total fat relative to their counterparts.

Our findings showed that recommended carbohydrate intake plus high MUFA intake presented the strongest protective association with CMD outcomes of any dietary pattern we investigated in this study. This is consistent with a meta-analysis conducted in 2017 that demonstrated lower fat mass and blood pressure associated with MUFA intake >12%en compared to a low MUFA intake group of ≤12%en and improved lipid profiles when MUFA intake was further increased to >15%en [39]. These categorizations are consistent with the MUFA stratifications of this study, ranging from <12.9%en to >14%en. Others found a reduction of android fat and diastolic blood pressure in groups receiving supplementation with high MUFA [40]. Among those that consumed restricted carbohydrate diets in our study, the opposite trend was observed, where high MUFA intake was associated with elevated odds of CMD. These divergent findings suggest that the health effects of MUFA intake may be moderated by carbohydrate intake, highlighting a research gap that calls for further study.

As CMD continues to present a major risk to health, understanding the influence of diet on CMD is critical [1]. This research has the potential to inform the development of future randomized controlled trials, which are needed to assess causal relationships between carbohydrate restriction and CMD risk when diets are stratified by fat intake. Our research raises questions for further study about low-carbohydrate diets and long-term health because of the strong associations we observed between restricted carbohydrate diets and prevalent CMD, CVD, CHD, and diabetes when compared with recommended carbohydrate diet groups, specifically when compared across fat stratifications.

This study has several strengths. To the best our knowledge, this is the first nationally representative study to investigate the association between restricted carbohydrate diets and prevalent CMD while stratifying by fat classes, which sheds light on the interactive effects of carbohydrate and fat intake on chronic health outcomes. Fat classes were measured comprehensively (as total fat) and separately (as SFA, MUFA, and PUFA) to evaluate fat classes with the strongest associations with health outcomes. Outcomes were measured comprehensively (as CMD) and separately (as CVD, CHD, stroke, and diabetes) based on standard clinical practice guidelines to further investigate heterogeneity in strength of association. All statistical models were adjusted for known sociodemographic and behavioral risk factors, and additional adjustments were made for dietary factors (foods and nutrients) with known and suspected association with CMD. Because the models for each dietary fat class (MUFA, PUFA, SFA) were adjusted for intakes of the 2 remaining fat classes, the results demonstrate the effects of each fat class without confounding that may result from high intakes of 2 or all fat classes simultaneously. Usual food and nutrient intakes were estimated from multiple 24-h recalls using the NCI method, which reduces bias by accounting for the variation that occurs within and between participants, measuring the intake of foods and nutrients consumed daily as well as episodically, and correlating the intake amount to the probability of intake [41]. Finally, the sampling design and large sample size make these findings generalizable to the US population.

This study also has some limitations. This was a cross-sectional study so causality cannot be inferred, and it is possible that participants adopted restricted carbohydrate diets after being diagnosed with CMD or other conditions. The duration of each of the participants’ diet practices could not be ascertained because of limited data availability, which may have introduced bias. Data on sociodemographics, lifestyle factors, dietary factors, and health status were merged from 1999–2018 to capture the long-term consumer focus on carbohydrate restricted diets and to increase statistical power, yet this may have obscured temporal patterns in diet-disease relationships. Statistical models accounted for a robust set of potential confounding variables, but residual confounding from unmeasured variables cannot be ruled out. Finally, self-reported dietary data are subject to measurement error including social desirability bias, in which participants alter reported consumption to improve perceived healthfulness. On the population level, however, self-reported dietary data can provide a valuable estimation of dietary intake [42].

In conclusion, this study of ∼20,000 adults demonstrated a 15% higher likelihood of CMD in those who consumed restricted carbohydrate diets compared with those that met daily macronutrient recommendations. Higher intake of saturated, monounsaturated, and polyunsaturated fat was associated with elevated likelihood of having CMD among those that consumed restricted carbohydrate diets. Robust scientific evidence demonstrates the cardiovascular benefits of dietary unsaturated fats (polyunsaturated and monounsaturated fats), particularly when they replace saturated and trans fats. Several factors, including food sources of PUFAs and potential simultaneous increase in trans or total fat, should be considered when interpreting these results. These findings suggest that more prospective research is needed to further examine the causal relationship between carbohydrate restriction and long-term health outcomes when stratifying by fat class.

## Author disclosures

ZC has research awards from The Thomas F. and Kate Miller Jeffress Memorial Trust, and the Commonwealth Center for Energy and the Environment at William & Mary, for projects unrelated to the present study; and received honoraria from MKYoung Food & Nutrition Strategies, National Geographic Society, Ohio State University, and *Nutrition Today* for professional activities unrelated to the present research. MAB has a research award from the United Soybean Board that has no relevance to the present project. MAB serves as a scientific advisor for Bath and Body Works and serves on the Board of Directors for ASN in roles that are unrelated to this project.

## Acknowledgments

ZC and MAB: conceived the study, designed the research, and obtained funding. CK: wrote the primary drafts of the manuscript. ZC and LKJ: performed data management and statistical analyses. All authors discussed the findings, interpretations, and co-wrote the final versions of the paper. All authors read and approved the final version of the manuscript.

## Data availability

Data described in the article, code book, and analytic code will be made available upon request.

## Appendix A Supplementary data

Multimedia components 1 Multimedia components 1
