# Supplementary material for: Fat Intake Modifies the Association between Restricted Carbohydrate Diets and Prevalent Cardiometabolic Diseases among Adults in the United States: National Health and Nutrition Examination Survey, 1999–2018
Source: Curr Dev Nutr. 2022 Dec 23;7(1):100019. doi: 10.1016/j.cdnut.2022.100019 (PMC10100922; doi:10.1016/j.cdnut.2022.100019)
Supplement: Multimedia components 1 [file mmc1.pdf]

Kowalski et al. Fat intake modifies the association between restricted carbohydrate diets and prevalent cardiometabolic diseases among adults in the United States: National Health and Nutrition Examination Survey, 1999-2018

Supplemental Table 1: Associations between carbohydrate intake and cardiometabolic diseases (n=19,078)

Outcome	n	OR (95% CI)[1]	P[2]
Cardiometabolic disease			
Restricted carbohydrate	4424	1.15 (1.14-1.16)	<0.001
Recommended carbohydrate	6628	1.02 (1.02-1.03)	<0.001
Recommended all macronutrients	8026	Reference	
Cardiovascular disease			
Restricted carbohydrate	4424	1.01 (1.00-1.02)	0.013
Recommended carbohydrate	6628	0.95 (0.94-0.96)	<0.001
Recommended all macronutrients	8026	Reference	
Coronary heart disease			
Restricted carbohydrate	4424	1.04 (1.03-1.05)	<0.001
Recommended carbohydrate	6628	0.91 (0.90-0.91)	<0.001
Recommended all macronutrients	8026	Reference	
Stroke			
Restricted carbohydrate	4424	0.94 (0.93-0.95)	<0.001
Recommended carbohydrate	6628	1.11 (1.10-1.12)	<0.001
Recommended all macronutrients	8026	Reference	
Diabetes			
Restricted carbohydrate	4424	1.19 (1.19-1.20)	<0.001
Recommended carbohydrate	6628	1.05 (1.05-1.05)	<0.001
Recommended all macronutrients	8026	Reference	

Restricted carbohydrate (<45%en carbohydrate); recommended carbohydrate (45-65%en carbohydrate); recommended all macronutrients (total carbohydrate, 45-65%en; total fat, 20-35%en; total protein, 10-35%en; α-linolenic acid, 0.6-1.2%en; and linoleic acid, 5-10%en).

[1]Adjusted for age, sex, education, race/ethnicity, income-to-poverty ratio, physical activity, smoking status, energy, refined grains, added sugars, fiber, protein, survey cycle, alcohol.

[2]Differences between diet categories assessed using Wald tests with $P<0.05$.

Supplemental Table 2: Associations between carbohydrate intake and cardiometabolic diseases, stratified by saturated fat intake (n=19,078)

Outcome	n	OR (95% CI)[1]	P[2]
Cardiometabolic disease			
Restricted carbohydrate			
High saturated fat[3]	1386	1.20 (1.19-1.22)	<0.001
Moderate saturated fat[4]	1481	1.15 (1.14-1.16)	<0.001
Low saturated fat[5]	1557	1.00 (0.98-1.01)	0.897
Recommended carbohydrate			
High saturated fat[3]	1165	1.02 (1.01-1.03)	<0.001
Moderate saturated fat[4]	2392	1.03 (1.03-1.04)	<0.001
Low saturated fat[5]	3071	1.02 (1.02-1.03)	<0.001
Recommended all macronutrients			
High/moderate saturated fat[3/4]	1412	1.11 (1.11-1.12)	<0.001
Low saturated fat[5]	6614	Reference	
Cardiovascular disease			
Restricted carbohydrate			
High saturated fat[3]	1386	1.05 (1.04-1.06)	<0.001
Moderate saturated fat[4]	1481	0.99 (0.98-1.00)	0.108
Low saturated fat[5]	1557	1.01 (1.00-1.03)	0.002
Recommended carbohydrate			
High saturated fat[3]	1165	0.92 (0.91-0.92)	<0.001
Moderate saturated fat[4]	2392	0.89 (0.89-0.90)	<0.001
Low saturated fat[5]	3071	1.03 (1.03-1.04)	<0.001
Recommended all macronutrients			
High/moderate saturated fat[3/4]	1412	1.05 (1.05-1.06)	<0.001
Low saturated fat[5]	6614	Reference	
Coronary heart disease			
Restricted carbohydrate			
High saturated fat[3]	1386	1.10 (1.08-1.12)	<0.001
Moderate saturated fat[4]	1481	1.03 (1.02-1.05)	<0.001
Low saturated fat[5]	1557	1.02 (1.00-1.04)	0.011

Recommended carbohydrate			
High saturated fat[3]	1165	0.88 (0.87-0.89)	<0.001
Moderate saturated fat[4]	2392	0.85 (0.85-0.86)	<0.001
Low saturated fat[5]	3071	0.99 (0.98-0.99)	<0.001
Recommended all macronutrients			
High/moderate saturated fat[3/4]	1412	1.04 (1.03-1.04)	<0.001
Low saturated fat[5]	6614	Reference	
Stroke			
Restricted carbohydrate			
High saturated fat[3]	1386	1.00 (0.99-1.02)	0.999
Moderate saturated fat[4]	1481	0.98 (0.97-0.99)	<0.001
Low saturated fat[5]	1557	0.93 (0.91-0.95)	<0.001
Recommended carbohydrate			
High saturated fat[3]	1165	1.15 (1.12-1.17)	<0.001
Moderate saturated fat[4]	2392	1.01 (1.00-1.02)	0.005
Low saturated fat[5]	3071	1.21 (1.20-1.21)	<0.001
Recommended all macronutrients			
High/moderate saturated fat[3/4]	1412	1.01 (0.99-1.03)	0.115
Low saturated fat[5]	6614	Reference	
Diabetes			
Restricted carbohydrate			
High saturated fat[3]	1386	1.17 (1.16-1.17)	<0.001
Moderate saturated fat[4]	1481	1.16 (1.14-1.17)	<0.001
Low saturated fat[5]	1557	0.99 (0.96-1.01)	0.212
Recommended carbohydrate			
High saturated fat[3]	1165	1.04 (1.03-1.05)	<0.001
Moderate saturated fat[4]	2392	1.10 (1.09-1.10)	<0.001
Low saturated fat[5]	3071	0.99 (0.98-0.99)	<0.001
Recommended all macronutrients			
High/moderate saturated fat[3/4]	1412	1.10 (1.09-1.10)	<0.001
Low saturated fat[5]	6614	Reference	

Restricted carbohydrate (<45%en carbohydrate); recommended carbohydrate (45-65%en carbohydrate); recommended all macronutrients (total carbohydrate, 45-65%en; total fat, 20-35%en; total protein, 10-35%en; α-linolenic acid, 0.6-1.2%en; and linoleic acid, 5-10%en).

[1]Adjusted for age, sex, education, race/ethnicity, income-to-poverty ratio, physical activity, smoking status, energy, refined grains, added sugars, fiber, %en protein, survey cycle, Body Mass Index, alcohol, %en total polyunsaturated fatty acids, %en total monounsaturated fatty acids

[2]Differences between diet categories assessed using Wald tests with $P<0.05$.

[3] High saturated fat (>12.5%en)

[4]Moderate saturated fat (11.4-12.5%en)

[5]Low saturated fat (<11.4%en)

Supplemental Table 3: Associations between carbohydrate intake and cardiometabolic diseases, stratified by monounsaturated fat intake (n=19,078)

Outcome	n	OR (95% CI)[1]	P[2]
Cardiometabolic disease			
Restricted carbohydrate			
High monounsaturated fat[3]	1512	1.16 (1.15-1.17)	<0.001
Moderate monounsaturated fat[4]	1535	1.08 (1.06-1.09)	<0.001
Low monounsaturated fat[5]	1377	0.87 (0.85-0.88)	<0.001
Recommended carbohydrate			
High monounsaturated fat[3]	612	0.88 (0.87-0.88)	<0.001
Moderate monounsaturated fat[4]	2783	0.98 (0.97-0.98)	<0.001
Low monounsaturated fat[5]	3233	1.01 (1.01-1.01)	<0.001
Recommended all macronutrients			
High/moderate monounsaturated fat[3/4]	478	0.81 (0.81-0.82)	<0.001
Low monounsaturated fat[5]	7548	Reference	
Cardiovascular disease			
Restricted carbohydrate			
High monounsaturated fat[3]	1512	1.04 (1.03-1.06)	<0.001
Moderate monounsaturated fat[4]	1535	1.18 (1.17-1.19)	<0.001
Low monounsaturated fat[5]	1377	0.82 (0.81-0.82)	<0.001
Recommended carbohydrate			
High monounsaturated fat[3]	612	0.92 (0.91-0.92)	<0.001
Moderate monounsaturated fat[4]	2783	0.97 (0.96-0.97)	<0.001
Low monounsaturated fat[5]	3233	0.98 (0.98-0.98)	<0.001
Recommended all macronutrients			
High/moderate monounsaturated fat[3/4]	478	0.86 (0.86-0.86)	<0.001
Low monounsaturated fat[5]	7548	Reference	
Coronary heart disease			
Restricted carbohydrate			
High monounsaturated fat[3]	1512	1.05 (1.03-1.06)	<0.001
Moderate monounsaturated fat[4]	1535	1.28 (1.26-1.29)	<0.001
Low monounsaturated fat[5]	1377	0.82 (0.80-0.83)	<0.001

Recommended carbohydrate			
High monounsaturated fat[3]	612	0.85 (0.84-0.86)	<0.001
Moderate monounsaturated fat[4]	2783	0.94 (0.94-0.95)	<0.001
Low monounsaturated fat[5]	3233	0.93 (0.93-0.93)	<0.001
Recommended all macronutrients			
High/moderate monounsaturated fat[3/4]	478	0.86 (0.86-0.87)	<0.001
Low monounsaturated fat[5]	7548	Reference	
Stroke			
Restricted carbohydrate			
High monounsaturated fat[3]	1512	1.07 (1.03-1.12)	<0.001
Moderate monounsaturated fat[4]	1535	0.95 (0.92-0.98)	<0.001
Low monounsaturated fat[5]	1377	0.67 (0.65-0.69)	<0.001
Recommended carbohydrate			
High monounsaturated fat[3]	612	1.04 (1.02-1.07)	<0.001
Moderate monounsaturated fat[4]	2783	1.06 (1.04-1.08)	<0.001
Low monounsaturated fat[5]	3233	1.18 (1.17-1.18)	<0.001
Recommended all macronutrients			
High/moderate monounsaturated fat[3/4]	478	0.93 (0.92-0.93)	<0.001
Low monounsaturated fat[5]	7548	Reference	
Diabetes			
Restricted carbohydrate			
High monounsaturated fat[3]	1512	1.12 (1.10-1.15)	<0.001
Moderate monounsaturated fat[4]	1535	0.98 (0.96-1.00)	0.01
Low monounsaturated fat[5]	1377	0.92 (0.89-0.95)	<0.001
Recommended carbohydrate			
High monounsaturated fat[3]	612	0.85 (0.84-0.86)	<0.001
Moderate monounsaturated fat[4]	2783	0.98 (0.97-0.98)	<0.001
Low monounsaturated fat[5]	3233	1.01 (1.00-1.01)	<0.001
Recommended all macronutrients			
High/moderate monounsaturated fat[3/4]	478	0.85 (0.84-0.85)	<0.001
Low monounsaturated fat[5]	7548	Reference	

Restricted carbohydrate (<45%en carbohydrate); recommended carbohydrate (45-65%en carbohydrate); recommended all macronutrients (total carbohydrate, 45-65%en; total fat, 20-35%en; total protein, 10-35%en; α-linolenic acid, 0.6-1.2%en; and linoleic acid, 5-10%en).

[1]Adjusted for age, sex, education, race/ethnicity, income-to-poverty ratio, physical activity, smoking status, energy, refined grains, added sugars, fiber, %en protein, survey cycle, Body Mass Index, alcohol, %en saturated fatty acids, %en polyunsaturated fatty acids.

[2]Differences between diet categories assessed using Wald tests with $P<0.05$.

[3]High monounsaturated fat (>14%en)

[4]Moderate monounsaturated fat (12.9-14%en)

[5]Low monounsaturated fat (<12.9%en)

Supplemental Table 4: Associations between carbohydrate intake and cardiometabolic diseases, stratified by polyunsaturated fat intake (n=19,078)

Outcome	n	OR (95% CI)[1]	P[2]
Cardiometabolic disease			
Restricted carbohydrate			
High polyunsaturated fat[3]	1477	1.11 (1.10-1.12)	<0.001
Moderate polyunsaturated fat[4]	1502	1.06 (1.05-1.07)	<0.001
Low polyunsaturated fat[5]	1445	0.98 (0.96-0.99)	<0.001
Recommended carbohydrate			
High polyunsaturated fat[3]	1524	1.04 (1.03-1.04)	<0.001
Moderate polyunsaturated fat[4]	2243	0.97 (0.96-0.97)	<0.001
Low polyunsaturated fat[5]	2861	0.95 (0.95-0.95)	<0.001
Recommended all macronutrients			
High/moderate polyunsaturated fat[3/4]	3259	0.99 (0.99-0.99)	<0.001
Low polyunsaturated fat[5]	4767	Reference	
Cardiovascular disease			
Restricted carbohydrate			
High polyunsaturated fat[3]	1477	1.02 (1.01-1.03)	<0.001
Moderate polyunsaturated fat[4]	1502	1.05 (1.04-1.06)	<0.001
Low polyunsaturated fat[5]	1445	0.92 (0.91-0.93)	<0.001
Recommended carbohydrate			
High polyunsaturated fat[3]	1524	0.94 (0.94-0.95)	<0.001
Moderate polyunsaturated fat[4]	2243	0.94 (0.93-0.94)	<0.001
Low polyunsaturated fat[5]	2861	0.93 (0.93-0.93)	<0.001
Recommended all macronutrients			
High/moderate polyunsaturated fat[3/4]	3259	0.93 (0.93-0.93)	<0.001
Low polyunsaturated fat[5]	4767	Reference	
Coronary heart disease			
Restricted carbohydrate			
High polyunsaturated fat[3]	1477	1.08 (1.06-1.09)	<0.001
Moderate polyunsaturated fat[4]	1502	1.16 (1.14-1.17)	<0.001
Low polyunsaturated fat[5]	1445	0.99 (0.98-1.01)	0.240

Recommended carbohydrate			
High polyunsaturated fat[3]	1524	0.98 (0.97-0.99)	<0.001
Moderate polyunsaturated fat[4]	2243	0.91 (0.90-0.92)	<0.001
Low polyunsaturated fat[5]	2861	0.91 (0.92-0.91)	<0.001
Recommended all macronutrients			
High/moderate polyunsaturated fat[3/4]	3259	0.99 (0.99-1.00)	<0.001
Low polyunsaturated fat[5]	4767	Reference	
Stroke			
Restricted carbohydrate			
High polyunsaturated fat[3]	1477	0.83 (0.82-0.84)	<0.001
Moderate polyunsaturated fat[4]	1502	0.84 (0.83-0.85)	<0.001
Low polyunsaturated fat[5]	1445	0.91 (0.90-0.92)	<0.001
Recommended carbohydrate			
High polyunsaturated fat[3]	1524	0.92 (0.92-0.93)	<0.001
Moderate polyunsaturated fat[4]	2243	0.99 (0.99-1.00)	<0.001
Low polyunsaturated fat[5]	2861	1.09 (1.09-1.10)	<0.001
Recommended all macronutrients			
High/moderate polyunsaturated fat[3/4]	3259	0.84 (0.84-0.85)	<0.001
Low polyunsaturated fat[5]	4767	Reference	
Diabetes			
Restricted carbohydrate			
High polyunsaturated fat[3]	1477	1.08 (1.07-1.09)	<0.001
Moderate polyunsaturated fat[4]	1502	1.05 (1.03-1.06)	<0.001
Low polyunsaturated fat[5]	1445	1.02 (1.00-1.05)	0.008
Recommended carbohydrate			
High polyunsaturated fat[3]	1524	1.10 (1.10-1.10)	<0.001
Moderate polyunsaturated fat[4]	2243	1.00 (1.00-1.01)	0.210
Low polyunsaturated fat[5]	2861	0.91 (0.91-0.91)	<0.001
Recommended all macronutrients			
High/moderate polyunsaturated fat[3/4]	3259	1.01 (1.01-1.02)	<0.001
Low polyunsaturated fat[5]	4767	Reference	

Restricted carbohydrate (<45%en carbohydrate); recommended carbohydrate (45-65%en carbohydrate); recommended all macronutrients (total carbohydrate, 45-65%en; total fat, 20-35%en; total protein, 10-35%en; α-linolenic acid, 0.6-1.2%en; and linoleic acid, 5-10%en).

1Adjusted for age, sex, education, race/ethnicity, income-to-poverty ratio, physical activity, smoking status, energy, refined grains, added sugars, fiber, %en protein, survey cycle, Body Mass Index, alcohol, %en total saturated fatty acids, %en total monounsaturated fatty acids.

[2]Differences between diet categories assessed using Wald tests with $P<0.05$.

[3]High polyunsaturated fat (>8.5%en)

[4]Moderate polyunsaturated fat (7.6-8.5%en)

[5]Low polyunsaturated fat (<7.6%en)

Supplemental Table 5: Associations between carbohydrate intake and cardiometabolic diseases, stratified by total fat intake (n=19,078)

Outcome	n	OR (95% CI)[1]	P[2]
Cardiometabolic disease			
Restricted carbohydrate			
High total fat[3]	1434	1.41 (1.39-1.43)	<0.001
Moderate total fat[4]	1538	1.09 (1.08-1.09)	<0.001
Low total fat[5]	1452	0.92 (0.90-0.93)	<0.001
Recommended carbohydrate			
High total fat[3]	254	1.15 (1.14-1.17)	<0.001
Moderate total fat[4]	2731	1.08 (1.07-1.09)	<0.001
Low total fat[5]	3643	1.00 (1.00-1.00)	<0.001
Recommended all macronutrients	8026	Reference	
Cardiovascular disease			
Restricted carbohydrate			
High total fat[3]	1434	1.06 (1.05-1.08)	<0.001
Moderate total fat[4]	1538	1.06 (1.05-1.07)	<0.001
Low total fat[5]	1452	0.81 (0.81-0.82)	<0.001
Recommended carbohydrate			
High total fat[3]	254	1.11 (1.10-1.12)	<0.001
Moderate total fat[4]	2731	0.92 (0.92-0.93)	<0.001
Low total fat[5]	3643	0.98 (0.98-0.98)	<0.001
Recommended all macronutrients	8026	Reference	
Coronary heart disease			
Restricted carbohydrate			
High total fat[3]	1434	1.08 (1.06-1.10)	<0.001
Moderate total fat[4]	1538	1.09 (1.07-1.10)	<0.001
Low total fat[5]	1452	0.87 (0.86-0.88)	<0.001
Recommended carbohydrate			
High total fat[3]	254	0.84 (0.83-0.86)	<0.001
Moderate total fat[4]	2731	0.90 (0.90-0.91)	<0.001
Low total fat[5]	3643	0.93 (0.93-0.93)	<0.001

Recommended all macronutrients	8026	Reference	
Stroke			
Restricted carbohydrate			
High total fat[3]	1434	0.99 (0.98-1.01)	0.380
Moderate total fat[4]	1538	1.08 (1.07-1.10)	<0.001
Low total fat[5]	1452	0.50 (0.49-0.51)	<0.001
Recommended carbohydrate			
High total fat[3]	254	1.78 (1.76-1.80)	<0.001
Moderate total fat[4]	2731	0.99 (0.98-1.00)	<0.001
Low total fat[5]	3643	1.17 (1.17-1.18)	<0.001
Recommended all macronutrients	8026	Reference	
Diabetes			
Restricted carbohydrate			
High total fat[3]	1434	1.50 (1.49-1.52)	<0.001
Moderate total fat[4]	1538	1.09 (1.08-1.09)	<0.001
Low total fat[5]	1452	0.95 (0.93-0.97)	<0.001
Recommended carbohydrate			
High total fat[3]	254	1.37 (1.35-1.38)	<0.001
Moderate total fat[4]	2731	1.16 (1.15-1.17)	<0.001
Low total fat[5]	3643	0.98 (0.98-0.98)	<0.001
Recommended all macronutrients	8026	Reference	

Restricted carbohydrate (<45%en carbohydrate); recommended carbohydrate (45-65%en carbohydrate); recommended all macronutrients (total carbohydrate, 45-65%en; total fat, 20-35%en; total protein, 10-35%en; α-linolenic acid, 0.6-1.2%en; and linoleic acid, 5-10%en).

[1]Adjusted for age, sex, education, race/ethnicity, income-to-poverty ratio, physical activity, smoking status, energy, refined grains, added sugars, fiber, %en protein, survey cycle, Body Mass Index, alcohol, unsaturated-to-saturated fat ratio.

[2]Differences between diet categories assessed using Wald tests with P<0.05.

[3] High total fat (>38.7%en)

[4]Moderate total fat (36.1-38.7%en)

[5] Low total fat (<36.1%en)
